# Cascaded-Cavity Fabry-Perot Interferometric Gas Pressure Sensor based on Vernier Effect

**DOI:** 10.3390/s18113677

**Published:** 2018-10-29

**Authors:** Peng Chen, Yutang Dai, Dongsheng Zhang, Xiaoyan Wen, Minghong Yang

**Affiliations:** National Engineering Laboratory for Fiber Optic Sensing Technology, Wuhan University of Technology, Luoshi Road 122, Wuhan 430070, China; chenpeng16@whut.edu.cn (P.C.); zhangdongsheng66@whut.edu.cn (D.Z.); wenxy@whut.edu.cn (X.W.)

**Keywords:** extrinsic Fabry-Perot interferometer, gas pressure, femtosecond laser, Vernier effect

## Abstract

An extrinsic Fabry-Perot interferometer (EFPI) composed of double fiber FP cavities in a glass capillary tube to generate Vernier effect has been fabricated and employed for gas pressure sensing. A lead-in single-mode fiber (LSMF) and a reflective single-mode fiber (RSMF) were inserted into the capillary tube to form a FP cavity. Femtosecond (fs) laser was used to ablate openings on a capillary tube for gas passage to the FP cavity. A fusion hole was also drilled on the end face of a SMF by fs laser. The sensitivity of the sensor is enhanced due to Vernier effect. Experimental results show that the sensitivity was as high as 86.64 nm/MPa in the range of 0~0.6 MPa, which is 32.8 times larger than that of an open-cavity EFPI sensor without Vernier effect. The temperature cross-sensitivity of the sensor was measured to be about 5.18 KPa/°C. The proposed sensor was characterized by its high sensitivity, compact structure and ease of fabrication, and would have extensive application prospects in gas sensing fields.

## 1. Introduction

Optic fiber sensors have been studied for few decades and attracted worldwide attention due to their advantages of immunity to electromagnetic, high temperature resistance, small size and light weight. Various optic fiber sensors were fabricated such as fiber Bragg grating (FBG) sensors [1,2], Mach-Zehnder interferometer (MZI) sensors [3,4], long-period grating (LPG) sensors [5,6] and Fabry-Perot interferometer (FPI) sensors [7,8], etc. Among them, FPI sensors are widely used in practical engineering applications owing to its simple principle, multiple manufacturing methods and convenience to combine with other fiber sensors [9,10,11]. Different kinds of FPI sensors based on diaphragms [12,13], photonic crystal fibers (PCFs) [14], and micro-structures [15] have been developed to measure gas pressure. Among them, diaphragm-based FPI sensors can provide ultra-high sensitivity through minimizing the diaphragm thickness by polishing, wet etching or selecting different diaphragm materials such as grapheme or silver [16,17,18,19]. An alternative method to obtain high sensitivity is a novel FPI sensors based on the Vernier effect, which is characterized with such qualities as low cost and being easy to implement [20,21,22,23,24].

Vernier caliper is a typical example of the Vernier effect used to improve the measurement accuracy. It contains two independent structures with different sizes. The overlap between the characteristic spectral lines of the two structures is used to perform the measurement. Therefore, we can make two FPI cavities with different free spectral ranges (FSRs) which were cascaded to generate Vernier effect. The interference spectrum of one cavity is changeable with environment, while that of the other stays the same. Therefore the composite spectrum will display interference fringes with a major peak and some minor peaks. The position of the major peak is located at the common resonant wavelength of the two cascaded FPIs. However, the cascaded FPI sensors with Vernier effect are mainly intrinsic which the interference spectrum not available until the sensors are finished [20,21,22,23,24]. So, in fact we are not sure if the sensor has a Vernier effect or the amplification factor of sensitivity is big enough to improve the accuracy.

In this paper, we developed a highly sensitive extrinsic Fabry-Perot interferometer (EFPI) gas pressure sensor by inserting a lead-in single-mode fiber (LSMF) and a reflective single-mode fiber (RSMF), from opposite ends, into a capillary tube. During the insertion, the interference fringes were monitored with an optical spectrum analyzer (OSA) to obtain Vernier effect. Additionally, higher sensitivity was achieved by controlling the distance between LSMF and RSMF, and also the period of the envelope. The sensor was experimentally demonstrated by measuring gas pressure up to 0.6 MPa. Through employing wavelength interrogation with a curve fitting method of superimposed interference fringes envelope [25,26], a gas pressure sensitivity of 86.64 nm/MPa was obtained, which is a dozen times improved compared with EFPI gas pressure sensors without Vernier effect [27].

## 2. Principle

**Figure 1:**
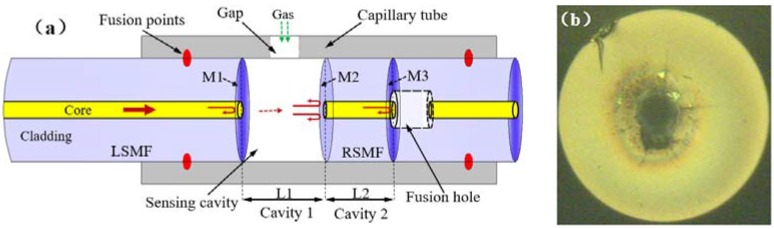
(**a**) Schematic of proposed sensor structure; (**b**) microscope image of fusion hole on the end face of fiber.

The schematic structure of the proposed all-fiber EFPI gas pressure sensor is shown in Figure 1. There are three reflective mirrors in the sensor, named by M1, M2, and M3, respectively. The air-cavity between M1 and M2 (cavity 1, the cavity length being L1) acts as sensing cavity since the ablated openings are located between them. The mirror of M2 and M3 form a silica-cavity with a length of L2 (cavity 2). M1 and M3 also form a cavity (cavity 3) with a length of L1 + L2, which is a hybrid cavity consisted of L1 and L2. The Vernier effect occurs once the FSR difference between cavity 1 and 2 is small. 

**Figure 2:**
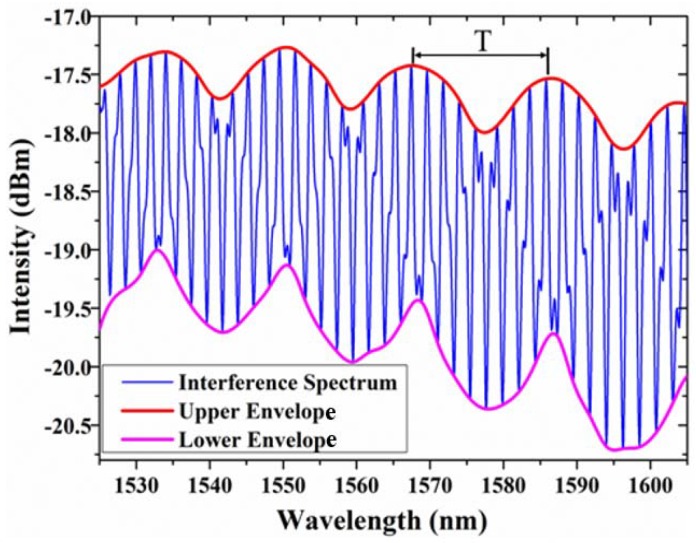
Interference spectrum of the proposed sensor with Vernier effect.

The beams reflected by the three mirrors will interfere when they come back into the LSMF. Supposing that the intensities of the reflected light from mirror 1, 2, 3 are $I_{1}$, $I_{2}$ and $I_{3}$, respectively, the intensity of the interference fringes could be expressed as:

(1) $$I=I_{1}+I_{2}+I_{3}+2\sqrt{I_{1}I_{2}}\cos(\phi_{12})+2\sqrt{I_{2}I_{3}}\cos(\phi_{23})+2\sqrt{I_{1}I_{3}}\cos(\phi_{13}),$$

where $\phi_{12}=4\pi n_{1}L_{1}/\lambda$, $\phi_{23}=4\pi n_{2}L_{2}/\lambda$ and $\phi_{13}=4\pi \left(n_{1}L_{1}+n_{2}L_{2}\right)/\lambda$ are the phase shifts of cavity 1, cavity 2 and cavity 3, respectively. λ is the wavelength, $n_{i}$ and $L_{i}$ are effect refractive index and length of the cavity *i* (*i* = 1, 2, 3). By Equation (1), we could find that the phase $\phi_{12}$ and $\phi_{13}$ change with the variation of $n_{1}$, so that the shift of the reflection spectrum is enlarged. The parameters of interference spectrum of Figure 2 are as follows: *n*_1_ = 1, *n*_2_ = 1.468, *L*_1_ = 527 µm, *L*_2_ = 404 µm and *λ* = 1550 nm. The *FSR* can be expressed as:

(2) $$FSR_{i}=\lambda^{2}/2n_{i}L_{i},$$

The calculated *FSR* for cavity 1 and cavity 2 are *FSR*_1_ = 2.279 nm and *FSR*_2_ = 2.025 nm. It assumes that the first peak of envelope is located at the common resonant wavelength of cavity 1 and cavity 2, an envelope period will be reached when two resonant wavelengths coincide again. The envelope period *T* is given by:

(3) $$T=FSR_{r}\times FSR_{s}/\left|FSR_{s}-FSR_{r}\right|,$$

where $FSR_{s}$, $FSR_{r}$ is the FSR of cavity 1 and cavity 2, respectively. As shown in Figure 2, the theoretical period with 18.169 nm agrees well with the actual measured envelope period of 18.40 nm. Noting that in practice the envelope period cannot be chosen larger than the available wavelength range of the measurement equipment.

The refractive index (RI) of air is related to temperature and pressure. Variation in pressure or temperature will introduce the change of RI of the air in the cavity [20]:

(4) $$n_{air}=1+7.82\times 10^{-7}P/\left(273.6+T\right),$$

where $n_{air}$, *P*, and *T* is RI of air, absolute pressure (Pa) and temperature (°C), respectively. It shows that $n_{air}$ increase linearly along with pressure when temperature is fixed. Change of $n_{air}$ would induce wavelength shift of cavity 1 by $\;\Delta \lambda =\lambda \cdot \left(\Delta n_{air}/n_{air}\right)$. Therefore, the major peak would shift to the adjacent minor peak and the test limit is given by [25]:

(5) $$\Delta n_{eff0}=n_{air}\left(FSR_{r}-FSR_{s}\right)/\lambda ,$$

In this way, the proposed EFPI sensor with Vernier effect has an ultra-high sensitivity *K* as decided by:

(6) $$K=FSR_{r}/\Delta n_{eff0}=\left(\lambda /n_{air}\right)\times \left(FSR_{r}/\left(FSR_{r}-FSR_{s}\right)\right)=K_{0}M,$$

where $M=FSR_{r}/\left(FSR_{r}-FSR_{S}\right)$ and $K_{0}=\lambda /n_{air}$. Here the *K*_0_ is the actual sensitivity of cavity 1. It is observed from Equation (6) that the sensitivity of the sensor with Vernier effect is *M* times higher than that of cavity 1. Besides, the envelope period is also *M* times higher than that of cavity 1 according to Equation (3). The actual *FSR*_1_ = 2.25 nm and the actual envelope period is 18.40 nm in Figure 2, so the amplification factor *M* = 8.18 agrees well with theoretical amplification factor *M* = 7.97. Equation (6) can also adapt at a certain temperature:

(7) $$K=\left(7.82\times 10^{-7}\Delta P/\left(273.6+T\right)\right)\times \left(FSR_{r}/\left(FSR_{r}-FSR_{s}\right)\right)=K_{P}M,$$

where Δ*P* is the change of gas pressure. Noting that for an increasing gas pressure *P*, the resonant wavelength of sensing cavity, that is cavity 1, will always shift to larger wavelength, however, the central wavelength of the envelope in reflection spectrum will shift to smaller wavelengths if $FSR_{s}>FSR_{r}$ and to larger wavelengths if $FSR_{s}<FSR_{r}$ [20,26].

Vernier effect can improve the sensitivity of sensor by decreasing the FSR difference of the two cavities and magnifying the envelope period. We can get as much magnification as possible by simply monitoring the size of envelope period in interference spectrum with OSA during sensor fabrication. The larger envelope period corresponds to a higher sensitivity.

However, in our sensor the interference spectrum intensity of cavity 3 was greater than that of the overlapping spectra of cavity 1 and cavity 2 at a same wavelength due to the large differences in phase, which generate some tiny peaks in the interference spectrum and change the shapes of the high-frequency fringes. If a specific fringe valley among the dips in the upper envelope is selected as the tracing location, a large error may exists due to the selected fringe valley is not the true central wavelength. The same situation applies to the lower envelope. In order to improve the detection precision, we introduced a signal processing method by connecting the peaks or valleys of high-frequency fringes to achieve the smoothing upper or lower envelope profile for sensing tracing (Figure 2). 

## 3. Experimental Results and Discussion

### 3.1. Sensor Fabrication

**Figure 3:**
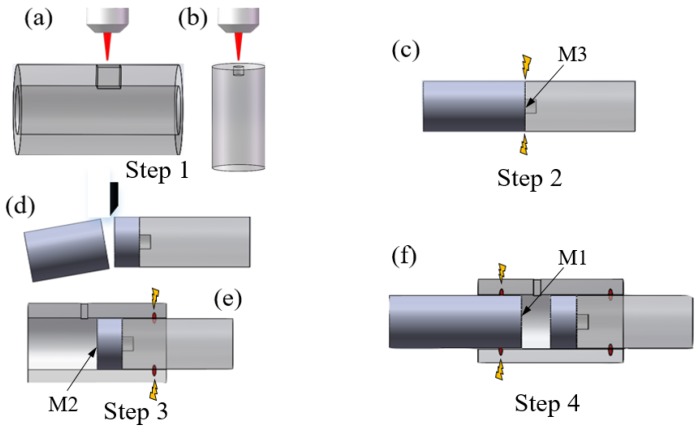
Schematic of the fabrication process of the EFPI gas pressure sensor: (**a**,**b**) micromachining of capillary tube and SMF; (**c**) fusion splicing of drilled SMF to an intact SMF; (**d**) cleaving of the spliced structure; (**e**) fusing with capillary tube; (**f**) inserting and fusing of another SMF (LSMF) in the capillary tube.

The detailed fabrication processes is illustrated by Figure 3. Firstly, a square opening (100 µm × 60 µm) on capillary tube and a fusion hole (20 µm in diameter) on the end face of single mode fiber (SMF) were fabricated by the fs laser (Figure 3a,b). The capillary tube had an external diameter of 250 µm and an internal diameter of 126 µm, and the diameter of SMF was 125 µm. The repetition rate, central wavelength, and pulse width of the fs laser was 1000 Hz, 780 nm and 180 fs, respectively. Considering the different mechanical strength of capillary tube and SMF, the laser pulse power was set to be 60 mW and 10 mW, respectively. Secondly, as shown in Figure 3c, an intact SMF was spliced to the drilled SMF to form a reflective mirror. During the process of splicing, lower discharge power and shorter discharge duration time (30 bit and 200 ms) were used to guarantee that the fusion hole was not completely collapsed. A silica-air interface (M3 in Figure 1) was formed at the right end facet of the intact SMF. Thirdly, facilitated by an optical microscope, the spliced intact SMF was cleaved to a certain length with the fiber cleaver (Fujikura, Tokyo, Japan, CT-30) as shown in Figure 3d, inserted and fused with capillary tube next to the gap by fiber fusion splicer (Ericsson, Stocholm, Sweden, FSU-975) as shown in Figure 3e. The arcing current was set to 12 mA and arcing duration time was 2 s. Herein an air-silica reflector (M2 in Figure 1) was produced at the other end of intact SMF. Thus this intact SMF containing two reflectors was also called RSMF. In the end, an LSMF was inserted from the other side of the capillary tube. During the insertion, interference spectrum was monitored in real time. Once we get the desired interference fringes with Vernier effect, the LSMF was hold in place, as shown in Figure 3f. The end of LSMF was also a silica-air reflector (M1 in Figure 1). 

### 3.2. Gas Pressure Measurement

**Figure 4:**
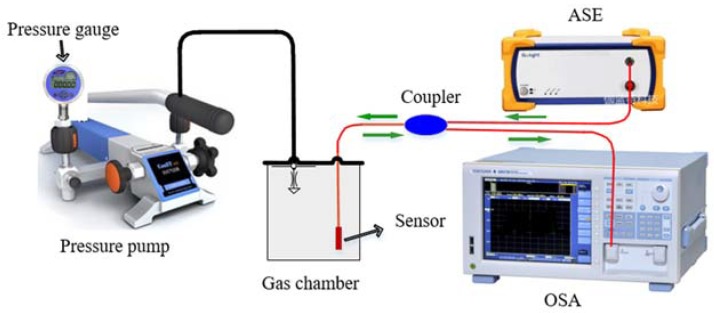
Experimental apparatus for the measure of gas pressure.

The experimental apparatus of gas pressure are illustrated in Figure 4. The sensing head was put in the gas chamber and the other end was connected to an amplified spontaneous emission (ASE, Ocean Optics, Largo, FL, USA) and an optical spectrum analyzer (OSA, AQ6370C, Yokogawa Electric Corporation, Tokyo, Japan) through a coupler. The broadband signal light was emitted from ASE with a spectrum region of 1525-1605 nm. The signal light was sent into the EFPI sensor, reflected back and detected by OSA. The resolution of OSA is 0.05 nm and the scan range is from 600 to 1700 nm. The gas pressure is generated by a pressure pump (ConST-162, ConST Ltd., Beijing, China) ranging from 0 to 0.6 MPa. A high-precision pressure gauge (ConST-211, ConST Ltd., Beijing, China) was introduced to measure the pressure with a precision of 0.001 MPa.

**Figure 5:**
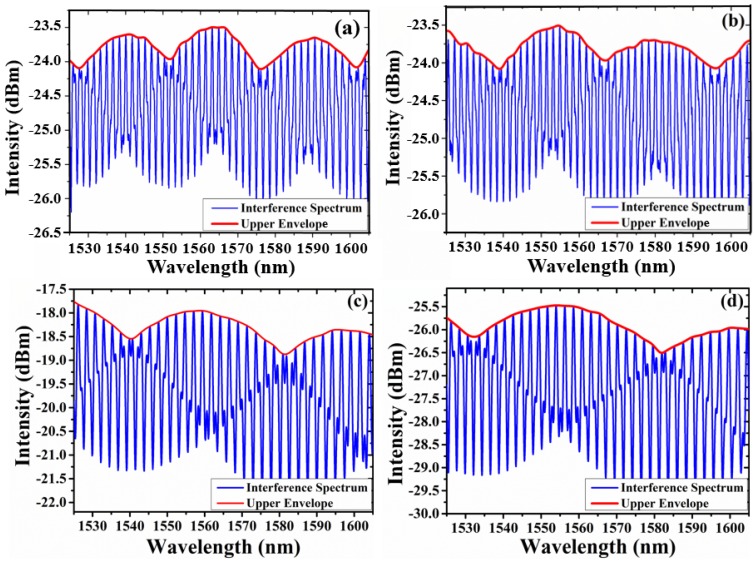
Interference spectrums of four sensors under 0 MPa: (**a**) sensor 1, (**b**) sensor 2, (**c**) sensor 3, (**d**) sensor 4.

Four open-cavity EFPI sensors with different envelope periods are fabricated. Table 1 lists the parameters of FSR of different sensors. Figure 5 shows the interference spectra of the four sensors under 0 MPa. Due to small differences between FSRs of cavity 1 and cavity 2, the sensors could generate Vernier effect clearly and periodic envelope signals were superposed on the sharp constituent peaks by connecting the peaks of high-frequency fringes. The envelope periods were 25.36 nm, 30.05 nm, 43.1 nm, 54.68 nm, respectively.

**Figure 6:**
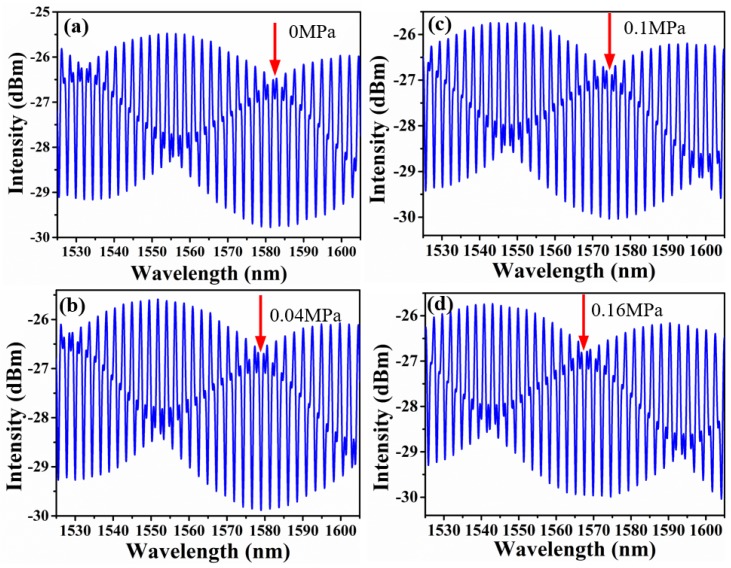
Output reflective spectra of sensor 4 with the relative pressure varying from 0 to 0.16 MPa: (**a**) 0 MPa; (**b**) 0.04 MPa; (**c**) 0.1 MPa; (**d**) 0.16 MPa.

To research the trait of the proposed sensor, sensor 4 was fixed at the gas chamber. The pressure of gas chamber was increased from 0 to 0.16 MPa with a step of 0.02 MPa, and maintained for 2 min at each step. Figure 6 presents the corresponding reflective spectra of sensor 4 under different gas pressure. It shows that the high-frequency fringes make little redshift, while the envelopes shift largely towards the short wavelength direction. According to Equation (6), when $FSR_{r}-FSR_{s}$ is negative, the sensitivity of the proposed sensor would be negative which means the spectrum shift towards the shorter wavelength direction with increased pressure or RI. Thus, the drift direction of high-frequency fringe was opposite to that of envelope. 

**Figure 7:**
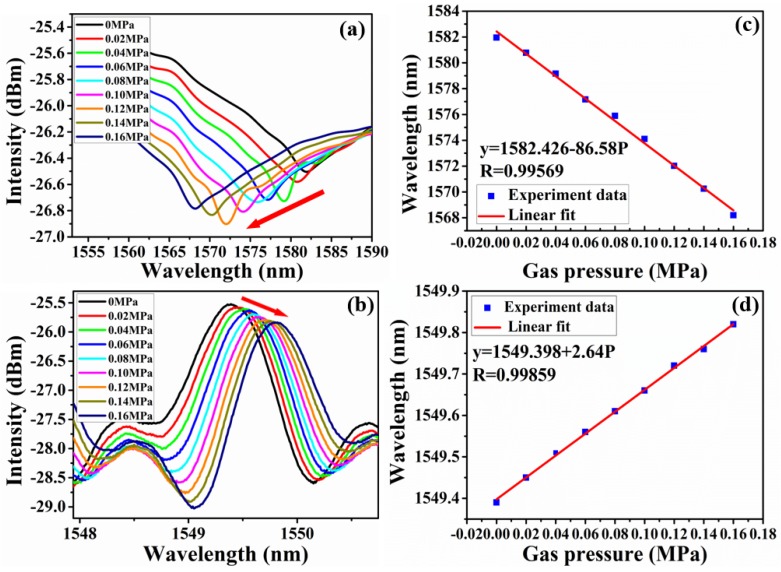
(**a**) The envelope curve of sensor 4 under different pressure; (**b**) partial enlarged drawing of high-frequency fringe under different pressure; (**c**) sensitivity of gas pressure measurement of sensor 4; (d) sensitivity of gas pressure measurement of high-frequency fringe.

Amplification effect of the proposed sensor was tested by comparing the envelope profile variation under different gas pressure. The dip at 1581.95 nm in black envelope profile was selected as the original tracing wavelength when the relative pressure difference is 0 MPa, and finally shifted to 1568.20 nm for 0.16 MPa change, corresponding to the wavelength change of 13.75 nm as shown in Figure 7a. Figure 7b shows the wavelength shift of the high-frequency fringes refer to cavity 1 at the wavelength of 1550 nm, the wavelength change of high-frequency fringes is only 0.43 nm. When gas pressure changes, the major peak would shift discretely and the wavelength shift of the major peak is equal to multiple FSRs of the two cascaded FPIs [25], that is the main reason that a slight wavelength shift of sensing cavity would lead a big change of spectra. The fitting curves of wavelength shift and pressure about envelope profile and high-frequency fringes are shown in Figure 7c,d, and both of them have good linear response. The sensitivity are −86.58 nm/MPa and 2.64 nm/MPa, respectively. The shift of envelope is *M* times higher than that of cavity 1 according to Equation (6), and the amplification factor is calculated to be *M* = 32.8. The theoretical amplification factor, *M* = 27.13, is a little smaller than the actual one, which may be caused by measurement errors. Besides, the wavelength resolution of OSA used in our experiment is 0.05 nm, so the gas pressure detection limit *ΔP* can be calculated as 0.58 KPa according to the quotient of wavelength resolution and sensor sensitivity.

**Figure 8:**
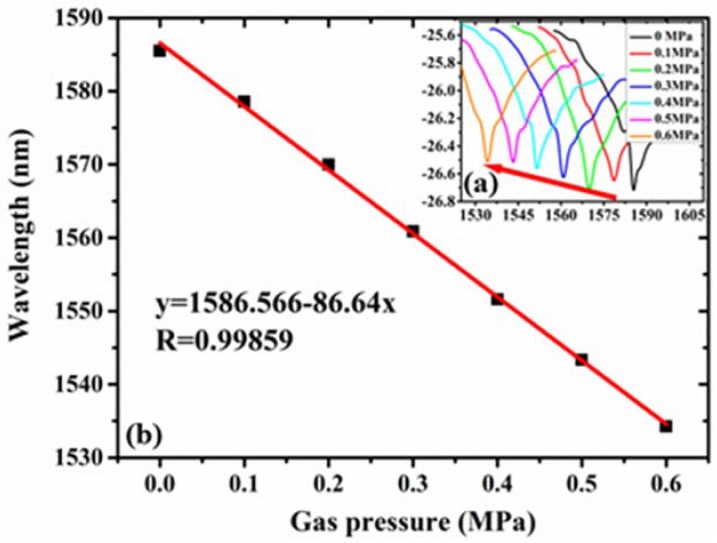
(**a**) Shifts of envelope for sensor 4 under different pressures; (**b**) wavelength versus gas pressure and the fitted line.

In order to test the consistency of the sensor under low- and high-pressure circumstance, the maximum pressure was increased to 0.6 MPa. The pressure was increased from 0 MPa to 0.6 MPa with a step of 0.1 MPa at room temperature, and stays of 2 min at each step. Figure 8a shows the envelope of sensor 4 at each step within one period. When the pressure increases, the interference fringes shifted to short wavelength. The fitting curve shown in Figure 8b indicated a sensitivity of 86.64 nm/MPa, which is consistent with the value under low pressure (86.58 nm/MPa).

**Figure 9:**
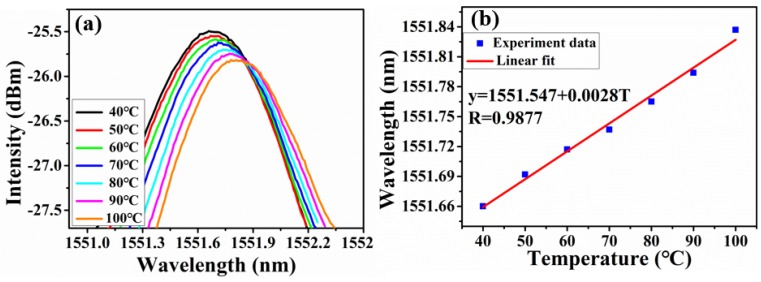
(**a**) Wavelength shift of the high-frequency fringe under different temperature; (**b**) calculated relative wavelength shift versus temperature.

**Figure 10:**
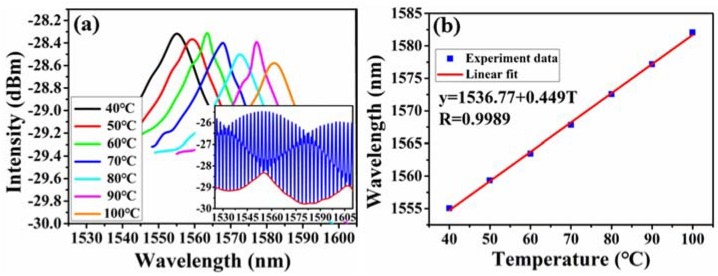
(**a**) Wavelength shift of the lower envelope under different temperature; (**b**) calculated relative wavelength shift versus temperature.

Temperature response of the proposed sensor was tested by putting it on the heating furnace with temperature accuracy of 0.1 °C. Temperature was increased gradually from 40 °C to 100 °C with a step of 10 °C. Through tracing the drift of high-frequency fringe, the temperature-induced wavelength shift was obtained, as shown in Figure 9. Then the sensitivity was calculated as 2.8 pm/°C by linear fitting. The lower envelope profiles under different temperature are also extracted in Figure 10a, corresponding to a temperature sensitivity of 449 pm/°C and the linearity is good, as shown in Figure 10b. The envelopes shift uniformly towards the longer wavelength, which is opposite to the direction of gas pressure-induced. It can be explained as following. The thermal expansion coefficient of fiber and capillary tube was different, the length change of cavity 2 was bigger than that of cavity 1 due to temperature variation. So cavity 2 now became the sensing cavity which cavity length is changeable, and cavity 1 was constant relative to cavity 2. Thus, the output interference spectrum of the sensor in pressure and temperature have difference in shift direction. Besides, the temperature amplification factor (160.35) was not in good agreement with the aforementioned theoretical (27.13). The reason is that both cavity 1 and cavity 2 have deformation with temperature variation, so the theoretical equation of amplification factor was no longer applicable. The temperature cross-sensitivity is calculated to be 5.18 KPa/°C, which is greater than minimum detectable gas pressure (0.58 KPa). Hence the sensor needs temperature compensation in practically for the best.

## 4. Conclusions

The fabrication process and gas pressure sensing performance of an EFPI sensor with Vernier effect have been demonstrated. Vernier effect is generated easily by controlling the distance between RSMF and LSMF, so that the gas pressure sensitivity could be improved significantly. Gas pressure can be monitored by measuring the shift of the spectrum envelope induced by the change of RI. Experimental results show that the sensor responds linearly to gas pressure. Gas pressure sensitivity as high as 86.64 nm/MPa was achieved, which is significantly increased compared with that without Vernier effect (2.64 nm/MPa). Besides, temperature cross-sensitivity of proposed sensor was 5.18 KPa/°C. All in all, it is a promising candidate for gas pressure sensing application in specified environment. 

## Figures and Tables

**Table 1:** Parameters of FSR of four sensors.

Parameter	Sensor 1	Sensor 2	Sensor 3	Sensor 4
*FSR*_s_ (nm)	2.113	2.033	2.091	2.025
*FSR*_r_ (nm)	1.960	1.920	2.005	1.953
